# US FDA–accelerated approvals and subsequent withdrawals: influence on Japanese clinical oncology practice guidelines

**DOI:** 10.1007/s10637-025-01524-9

**Published:** 2025-04-03

**Authors:** Hayase Hakariya, Akihiko Ozaki, Tetsuya Tanimoto

**Affiliations:** 1 Interfaculty Institute of Biochemistry, University of Tuebingen, 72076 Tuebingen, Germany; 2 Institute for Pharmaceutical and Social Health Sciences, Ise, Japan; 3 Breast and Thyroid Center, Jyoban Hospital of Tokiwa Foundation, Iwaki, Fukushima 972-8322 Japan; 4 Medical Governance Research Institute, Minato, Tokyo 108-0074 Japan; 5 Navitas Clinic, Tokyo, 190-0023 Japan

**Keywords:** Guideline recommendations, The US food and drug administration, Accelerated approval, Pharmaceutical and medical devices agency, Regulatory agency, Japan, Objective response rate, Progression-free survival

## Abstract

The US (US) Food and Drug Administration (FDA)-accelerated approval pathway facilitates early access to oncology drugs based on surrogate endpoints, with required confirmatory post-marketing trials. However, regulatory decisions vary globally, with some drugs withdrawn in the US remaining approved in Japan. We conducted a cross-sectional analysis of Japanese professional society guidelines, evaluating recommendations for seven accelerated approval cancer drugs withdrawn from the US market but retained in Japan. We assessed for level of evidence and level of treatment preference ratings with consensus across guidelines issued by the corresponding Japanese professional societies. Four of the seven drugs (57%) were recommended as highly or moderately preferred treatment options in Japanese guidelines: gemtuzumab ozogamicin for acute myeloid leukemia, gefitinib for EGFR-positive non-small cell lung cancer, bevacizumab for HER2-negative metastatic breast cancer, and atezolizumab with nab-paclitaxel for PD-L1-positive triple-negative breast cancer. Detailed analysis of regulatory history and background of guideline recommendation revealed discrepancies in the assessment of clinical benefits: gemtuzumab ozogamicin failed to demonstrate benefits amid safety concerns, while gefitinib, bevacizumab, and atezolizumab were more controversial, although they did not demonstrate improved overall survival in post-marketing trials. Despite regulatory withdrawal in the US due to unproven clinical benefits, drugs retained in Japan received positive guideline recommendations. This finding highlights regional variations in regulatory decisions and different approaches to benefit-risk assessments, suggesting a need for improved transparency in Japan’s regulatory decisions and guideline recommendations, with clearer justifications for endorsing drugs that are considered to have unproven clinical benefits in the US.

## Introduction

The accelerated approval pathway of the United States (US) Food and Drug Administration (FDA) allows the approval of novel oncologic drugs for patients with serious conditions and with unmet needs majorly based on surrogate or intermediate endpoints such as objective response rate (ORR) and sometimes progression-free survival (PFS) that are reasonably predictive of clinical benefit. After the accelerated approval, manufacturers must complete post-marketing confirmatory trials within a specified timeframe to verify the anticipated benefits. However, the accelerated approval program faces challenges, such as delays in drug manufacturers’ completion of confirmatory studies [1, 2], high prices despite uncertain clinical benefit [3], and slow withdrawal process when confirmatory post-marketing trial failed to prove clinical benefit [4, 5].

As many countries reference FDA decisions for their regulatory policies, a significant challenge arises from delays in US regulatory decisions due to prolonged or even non-completed post-marketing studies [5–7], which in turn has global implications [8]. Although FDA decision is not a gold standard of clinical evidence, this or a similar situation applies even in countries with their own regulatory bodies such as European countries and Japan [8, 9]. Japan has introduced a similar regulatory pathway of a US-accelerated approval, conditional early approval (CEA) in 2017 [10], albeit without the requirement for post-marketing confirmatory trials. Instead, the Japanese government endorses the importance of real-world data to substantiate the anticipated benefit [9, 11–13].

Indeed, cancer drug indications approved through accelerated approval but withdrawn from the US market have remained approved in Europe (eight cases) and Japan (seven cases) as of April 2023 [14, 15]. Of note, more than half of them 63% (5/8) in Europe and 71% (5/7) in Japan were withdrawn from the US market due to the unmet clinical endpoint in the post-marketing study such as PFS or overall survival (OS) [14].

Thus, investigating whether these drugs are recommended in clinical oncology practice guidelines is crucial, as professional society guidelines inform patient and physician decision-making. Indeed, the US treatment guidelines provided a high level of endorsement for accelerated approval drugs that have failed post-approval trials, sometimes even after the approval has been withdrawn or revoked [5]. Herein, we undertook a cross-sectional analysis of Japanese professional society guidelines to assess their recommendations for the aforementioned seven cancer drugs as of August 2024 [14].

## Methods

Although the Japan Society of Clinical Oncology (JSCO) comprehensively curates guidelines from professional societies from each field, Japan lacks a centralized guideline issuer; instead, each professional society issues its own guidelines using distinct criteria to determine levels of evidence and treatment preference rating through consensus, primarily based on the Grading of Recommendations, Assessment, Development and Evaluation (GRADE) approach [16, 17]. To standardize these criteria, we defined overall ratings for level of evidence and consensus (*A* to *D*), and treatment preference (*highly preferred* to *not recommended*) (Table 1). These guideline recommendations were issued by the Japanese Society of Hematology (JSH), the Japan Lung Cancer Society (JLCS), or the Japanese Breast Cancer Society (JBCS) [18–20]. We referenced the latest online guidelines from these societies, updated in 2022 or 2023, as of August 2024. Each drug-indication pair was assessed for level of evidence and level of treatment preference ratings based on these standardized ratings. As a reference, the US's National Comprehensive Cancer Network (NCCN) ratings are also listed in Table 1 [21]. We note that drugs with Japanese guideline recommendations do not necessarily be covered by national health insurance, and guidelines do not affect drug funding.

**Table 1:** Categories of level of evidence and consensus and treatment preference ratings in Japanese academic societies

NCCN rating [21]	NCCN Definition*	JSH rating† [18]	JLCS rating‡ [19]	JBCS rating‡ [20]	overall ratings
Level of evidence and consensus					
1	High level evidence with uniform panel consensus (> 85% of votes)	1	A	A	A
2A	Lower level of evidence with uniform panel consensus (> 85% of votes)	2A	B	B	B
2B	Lower level of evidence without uniform panel consensus (50–85% of votes)	2B	C	C	C
3	Any level of evidence but with major disagreement (25–50% of votes)	3.4	D	D	D
Treatment preference					
Preferred	Based on superior efficacy, safety, and evidence and, when appropriate, affordability	1	preferred	1	Highly Preferred
Alternative preferred	No definition given	2A	recommended	2	Moderately Preferred
Other recommended	May be somewhat less efficacious, more toxic, or based on less mature data; or significantly less affordable for similar outcomes	2B	recommend not to perform	3	Other recommended
Useful in certain circumstances	May be used for select patient population defined with recommendation	3.4§	prefer not to perform	4	Not recommended

*NCCN* National Comprehensive Cancer Network, *JSH* the Japanese society of hematology, *JLCS* the Japan lung cancer society, *JBCS* the Japanese breast cancer society, *Minds* Medical Information Distribution Service, *GRADE* Grading of Recommendations, Assessment, Development and Evaluation

^*^NCCN rating definition does not perfectly apply to each Japanese professional society’s guideline

^†^Level of evidence is assessed based on the Levels of Evidence for Adult and Pediatric Cancer Treatment Studies (PDQ®), known as National Cancer Institute-Physician Data Query (NCI-PDQ), where evidence is evaluated using binary scale: quality of research design and quality of study endpoint. Treatment preference is majorly based on NCCN guideline

^‡^JLCS and JBCS evidence ratings are based on Minds, a Japanese guideline center, and their treatment preference ratings majorly on GRADE

^§^Category 4 is provided to clarify that the treatment is negative recommendation for general clinical practice only when there is a unified consensus. This category is not intended to prohibit the treatment implementation in a clinical trial, as long as it adheres an appropriate research design and ethical guidelines

## Results

For each drug-indication pair, Table 2 shows treatment preference ratings, levels of evidence, consensus rates (%), year of guideline update, guideline issuer, and the dates of US withdrawal and approval in Japan. These drugs were indicated either for hematological, lung, or breast cancer, with the earliest approval in Japan being gemutuzumab ozogamicin in 2005 and the most recent atezolizumab in 2019. The median time from Japanese approval to US withdrawal was 2.2 years, ranging from 0.3 to 6.8 years. We discovered that four out of the seven drugs (57%) were highly or moderately preferred as treatment options with evidence levels of A or B (Table 2). To better understand why these four drugs have been recommended by Japanese professional societies, we scrutinized guidelines further, alongside with regulatory consequences of each drug in Japan and the US.

**Table 2:** Cancer drug indications with accelerated approval in the US that were withdrawn by the FDA but remain approved by the PMDA, and their guideline recommendations in Japan

Drug	Indication	Category of treatment preference ratings	level of evidence	consensus rate (%)	Year of Guideline Update	Guideline issuer [18–20]	Unmet Primary Endpoint or Trial Outcome Leading to Drug Withdrawal by the DFA	Date of approval in Japan (mm/dd/yyyy)	Date of approval in US (mm/dd/yyyy)
Gemtuzumab ozogamicin	AML	Moderately preferred	B	n.a	2023	JSH	OS at Phase 3 and safety concerns	5/26/2005	11/28/2011
Gefitinib*†	NSCLC	Moderately preferred	A	79% (22/28)	2023	JLCS	OS at Phase 3	10/21/2011	4/25/2012
Bevacizumab	HER2(-) breast cancer	Moderately preferred	A	97% (34/35)	2022	JBCS	No-reproducability in PFS and OS was not beneficial	7/14/2011	11/18/2011
Fludarabine phosphate	B-cell CLL	n.a	n.a	n.a	2023	JSH	Unable to complete confirmatory trial	10/2/2009	12/31/2011
Romidepsin	PTCL	n.a	n.a	n.a	2023	JSH	PFS at phase 3	5/30/2017	7/30/2021
Panobinostat‡	MM	n.a	n.a	n.a	2023	JSH	Unable to complete confirmatory trial	5/28/2015	3/24/2022
Atezolizumab	PD-L1( +) TNBC	Highly preferred	B	94% (33/35)	2022	JBCS	PFS at phase 3	08/07/2019	10/6/2021

*AML* acute myeloid leukemia, *HER2* human epidermal growth factor receptor 2, *MM* multiple myeloma, *NSCLC* non-small cell lung cancer, *PDL1* programmed death-ligand 1, *PTCL* peripheral T-cell lymphoma, *TNBC* triple-negative breast cancer, *JSH* the Japanese Society of Hematology, *JLCS* the Japan Lung Cancer Society, *JBCS* the Japanese Breast Cancer Society, *OS* overall survival, *PFS* progression-free survival, *PS* performance status

^*^Indication recommended in the JLCS guideline differs from the one in the US. Treatment in combination with carboplatin plus pemetrexed is recommended in Japan whereas US originally indicated the treatment as a monotherapy after failure of platinum-based and docetaxel chemotherapy

^†^This Japanese guideline recommendation is for patients with PS 0–1, and the recommendation differs between PS level. PS 0–1 was chosen in this study, as a key clinical trial for gefitinib approval (both in Japan and the US’s re-approval) involved 93% of patients with PS 0–1

^‡^Market sale of the panobinostat in Japan was suspended on March 2024 by the sponsor due to an undisclosed reason

### Gemtuzumab ozogamicin

Gemtuzumab ozogamicin for acute myeloid leukemia initially received US accelerated approval for treatment of 60 years or older patients with relapsed CD33‐positive and not candidates for cytotoxic chemotherapy [22]. This was later withdrawn from the US in 2011 due to its failure to prove clinical benefit among the safety concerns [23]. During the post-marketing trial, the US’s original regimen of 9 mg/m^2^ for two doses at least 14 days apart turned out to possess risks of fatal hepatotoxicity and veno‐occlusive disease (VOD), which were added as boxed warnings. However, this drug was re-approved in 2017 with a different patient subpopulation and a dose regimen of 3 mg/m^2^ days 1, 4, and 7. Whereas in Japan, the drug was approved in 2005 for relapsed or refractory CD33-positive AML with the US’s original regimen of 9 mg/m^2^ for two doses at least 14 days apart has been applied, indicating risks underlying the prescription. However, the Japanese regulatory authority and the professional society recommend gemtuzumab ozogamicin for patients who are not candidates for cytotoxic chemotherapy, specifying that the following regulatory-approved dose regimen—9 mg/m^2^ for two doses at least 14 days apart—should be used, despite being the higher-risk regimen that was withdrawn in the US [24].

### Gefitinib

Gefitinib was originally granted US accelerated approval in 2003 as monotherapy for locally advanced or metastatic NSCLC after the failure of platinum-based and docetaxel chemotherapy, but was withdrawn from the US market in 2012 due to the failure to improve OS at post-marketing phase 3 study. This drug was later re-approved for EGFR-positive patients in 2015, where randomized trials showed improved ORR and PFS [25, 26]. Given that the Japanese guideline recommends gefitinib only for epidermal growth factor receptor (EGFR)-positive patients, the recommendation is consistent with the later US regulatory decision. However, both Japan’s guideline recommendation and the US’s re-approval are based on the surrogate endpoint of ORR and PFS [27, 28], which may necessitate further study to prove improved OS.

### Bavacizumab

Bevacizumab in combination with paclitaxel for patients who have not received chemotherapy for metastatic HER2 negative breast cancer received the US-accelerated approval in 2008 and was withdrawn in 2011 due to the failure to improve OS, though it improved the PFS [29–31]. JBCS’s moderately preferred recommendation is based on their own meta-analysis, including nine randomized controlled trials (RCT), based on which they evaluated the OS as the most important outcome, and PFS, QoL, and toxicity as secondary outcomes. The society presumably recommended the treatment due to the PFS elongation, and they recognized that it does not improve OS. The indication has standard marketing authorization in Europe [15], and the therapeutic value of bevacizumab has been globally controversial [31].

### Atezolizumab

JBCS guideline recommends atezolizumab in combination with nab-paclitaxel, nanoparticle albumin–bound paclitaxel, as a highly preferred treatment against locally advanced or metastatic triple-negative breast cancer whose tumors express PD-L1. Its highly preferred recommendation by the JBCS is based on the single clinical trial IMpassion130, where they confirmed 7.5 months of OS improvement [32]. Whereas the drug was withdrawn from the US market on the basis of the following clinical trial IMpassion131, where they failed to show improvement of both PFS and OS [33]. However, atezolizumab was used in combination with paclitaxel (but not with nab-paclitaxel) in this study. Thus, the Japanese guideline might be appropriate on the basis of the result of IMpassion130; nonetheless, the clinical effect of atezolizumab remains inconclusive.

In contrast, the remaining three drugs—fludarabine phosphate for B-cell chronic lymphocytic leukemia (withdrawn from the US in 2011), romidepsin for peripheral T-cell lymphoma (withdrawn from the US in 2021), and panobinostat for multiple myeloma (withdrawn from the US in 2021)—were not listed in the Japanese guideline recommendations.

## Discussion

As previously documented [14, 15, 34, 35], regulatory approval decisions for drug indications through expedited programs show significant regional variations among the US, Europe, and Japan. The seven drugs examined in this study highlight these controversial discrepancies, where medications withdrawn in one region remain approved and recommended in another. Specifically, bevacizumab and atezolizumab represent such inconclusive cases among the four out of seven drugs (57%) that were highly or moderately preferred as treatment options in Japanese professional guidelines. However, based on our investigation, the judgments made by each regulatory authority appear generally acceptable, as the observed discrepancies largely depend on which endpoints were evaluated or what safety levels the evaluators were willing to tolerate.

Unlike the FDA’s accelerated approval pathway, which necessitates post-marketing confirmatory trials and allows drug withdrawals when clinical benefits are not confirmed, Japan’s regular (traditional) approval system, applied for most drugs, lacks a functional withdrawal pathway as of January 2025. More precisely, while this system technically exists in Japan, it rarely operates effectively, with regulators preferring to revise package inserts, limit indications, or strengthen warnings instead. This discrepancy in the regulatory attitude may be reflected in the case of gemtuzumab ozogamicin for acute myeloid leukemia, which on one hand was once withdrawn from the US market due to the efficacy and safety concerns, but remained approved in Japan with the same indication and dosages. Given the negative results from the post-marketing clinical trial, the Japanese authority should better explain and justify the rationale behind different regulatory conclusions.

This case additionally highlights that the medication is likely chosen in clinical practice in Japan, based on the guideline recommendation as a moderately preferred treatment option. While our findings do not discourage clinicians from prescribing these medications in specific contexts, this case underscores the potential need for refinement and optimization of recommendations for drugs with unproven clinical benefits, the selection of clinical questions and members, and overall structure. At the very least, they should elaborate on the rationale behind their guideline recommendation with such drugs. Introducing a checklist for good reporting of practice guidelines may also help [36].

Our study also highlighted the broader implications of differing regulatory and clinical practices across countries. International collaboration and harmonization on evidence evaluation may ultimately improve global standards for patient care and safety.

This study has limitations. First, our analysis focused exclusively on seven cancer drug-indication pairs initially approved under the FDA’s accelerated approval pathway, subsequently withdrawn from the US market, but retained in Japan. The sample number is limited, and our findings are not generalizable to drugs in other therapeutic areas or regulatory pathways. Second, our analysis did not reflect the discrepancy in regulatory stance. For instance, bevacizumab against breast cancer has been authorized in Europe, not only in Japan. This case highlights either cancer patients in the US may no longer have access to beneficial medications, as judged by European or Japanese regulatory standards, or patients in Europe or Japan might be receiving treatments with medications that lack clinical benefits, as judged by the US regulatory standards [14, 15, 37]. Lastly, while we standardized criteria for comparing evidence levels and treatment preferences ratings across guidelines, these simplifications may not capture all nuanced considerations. Cancer types are heterogeneous, and our approach may overlook clinical or molecular characteristics influencing recommendations.

## Data Availability

The datasets generated during and/or analyzed during the current study are available from the corresponding author on reasonable request.
